# Apoptosis-related microRNA-145-5p enhances the effects of pheophorbide a-based photodynamic therapy in oral cancer

**DOI:** 10.18632/oncotarget.17059

**Published:** 2017-04-12

**Authors:** Sook Moon, Do Kyeong Kim, Jin Kim

**Affiliations:** ^1^ Department of Dental Hygiene, College of Nursing Healthcare, Sorabol College, Gyeongju 38063, Republic of Korea; ^2^ Oral Cancer Research Institute, Department of Oral Pathology, Yonsei University College of Dentistry, Seoul 03722, Republic of Korea; ^3^ BK21 PLUS Project, Yonsei University College of Dentistry, Seoul 03722, Republic of Korea

**Keywords:** microRNA, oral cancer, pheophorbide a, photodynamic therapy, phototoxicity

## Abstract

MicroRNAs (miRNAs) regulate key biological processes, and their aberrant expression has been related to cancer development. Photodynamic therapy (PDT) has emerged as one of the most promising modalities for cancer treatment. However, the application of PDT has been limited to superficially localized human cancerous and precancerous lesions. To increase the usefulness of both PDT and miRNAs in cancer therapy, this study investigated whether apoptosis-related miRNA expression is influenced by PDT in oral cancer and whether miRNAs can enhance PDT efficacy. To achieve this goal, we performed a miRNA array-based comparison of apoptosis-related miRNA expression patterns following PDT using pheophorbide a (Pa) as a photosensitizer. After Pa-PDT, 13.1% of the miRNAs were down-regulated, and 16.7% of the miRNAs were up-regulated. Representative miRNAs were selected according to expression difference: miR-9-5p, miR-32-5p, miR-143-3p, miR-145-5p, miR-192-5p, miR-193a-5p, miR-204-5p, miR-212-3p, miR-338-3p, and miR-451a. Among them, only miR-145-5p showed the consistent reduction repeatedly in all cell lines after Pa-PDT. Further, the combined treatment of a miR-145-5p mimic and Pa-PDT increased phototoxicity, reactive oxygen species generation, and apoptotic cell death, suggesting that miRNAs expression could be a useful marker for enhancing the therapeutic effect of Pa-PDT. This study will provide a promising strategy for introducing miRNA as cancer therapy.

## INTRODUCTION

MicroRNAs (miRNAs, miRs) are a class of highly-conserved, short, non-coding RNAs that have emerged as important mediators of translational control. By causing translational arrest, mRNA cleavage or a combination of the two, miRNAs regulate the expression of their target genes, mostly via direct targeting of the 3’-untranslated region (UTR) of mRNAs [1–3]. Additionally, miRNAs regulate a wide range of biological processes, including apoptosis, differentiation and cell proliferation [4, 5]. Aberrant expression and function of miRNAs have been reported in many types of cancers [6, 7]. The pattern of miRNA expression varies dramatically across tumor types, and miRNA profiles reflect the developmental lineage and differentiation state of a tumor [8]. Moreover, miRNAs might enhance chemosensitivity and radiosensitivity in human cancer therapy [9, 10].

Photodynamic therapy (PDT) has received considerable attention as an alternative treatment modality in human cancer [11]. In particular, it is widely accepted that PDT has therapeutic effects on melanoma, leukemia, hepatocellular carcinoma, and oral squamous cell carcinoma (OSCC) [12–15]. The merits of PDT include selective treatment of disease sites, simple surgical procedure, and minimal damage. PDT includes two individually non-toxic components: light and a photosensitizer. Photosensitizers are taken up by tumor cells and are subsequently activated by light, producing reactive oxygen species (ROS) and causing cell death [16]. However, PDT has only shown limited success because of unanticipated side effects and insufficient tumor selectivity. Because of these shortcomings, clinical adoption of PDT has been partially successful only on superficially located cancers such as skin cancer or OSCC.

Considering that miRNAs facilitate chemosensitivity and radiosensitivity in cancer treatment, this study attempted to investigate whether miRNA expression is influenced by PDT and whether miRNAs can enhance the therapeutic effect of PDT. To achieve this goal, apoptosis-related miRNAs were analyzed in oral cancer after PDT using pheophorbide a (Pa) as a photosensitizer. We found that miRNA expression was altered by Pa-PDT. In particular, expression of miR-145-5p, an apoptosis-related miRNA, was markedly reduced, and treatment with a miR-145-5p mimic enhanced phototoxicity. This study demonstrates the feasibility of this novel approach introducing miRNA expression as a marker for enhancing the efficacy of PDT in cancer therapy.

## RESULTS

### Profiling of apoptosis-related miRNA expression by Pa-PDT

**Figure 1:**
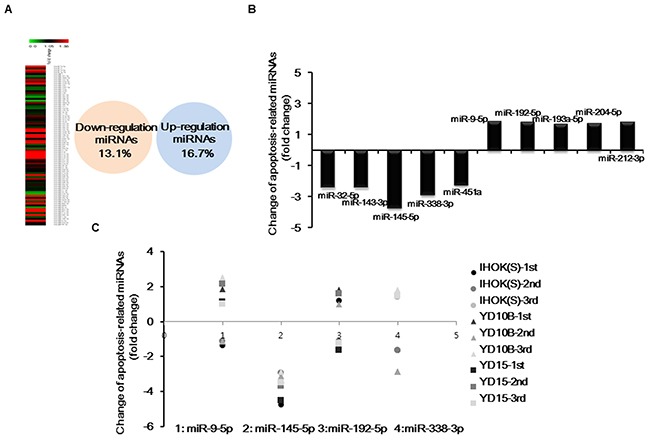
Apoptosis-related miRNAs expression profiling by Pa-PDT (A) Cells were incubated with 0.3 μM Pa for 2 h in a dark room Cells were then rinsed with PBS and irradiated by light (0.5 J/cm^2^). Hierarchical clustering of miRNA expression obtained with miScript miRNA microarrays and a Venn diagram of miRNA expression depicted the down-regulation and up-regulation of miRNAs by Pa-PDT. (**B**) Representative (top five) down-regulated and up-regulated miRNAs in YD10B. (**C**) Change in expression of representative miRNAs down-regulated and up-regulated by Pa-PDT in several cell lines was confirmed using quantitative real-time PCR.

To investigate whether miRNA expression is influenced by Pa-PDT, 84 apoptosis-related miRNAs were profiled in YD1OB OSCC cells using human microRNA arrays and quantitative PCR analysis. After treatment with Pa-PDT, 13.1% of the miRNAs were down-regulated, while 16.7% of the miRNAs were up-regulated compared to untreated cells (Figure 1A). We found that miR-145-5p was predominantly down-regulated (Figure 1B). To confirm these data, we selected 4 miRNAs showing the greatest up-regulation and down-regulation and repeated quantitative real-time PCR with 3 OSCC cell lines. Only miRNA-145-5p was consistently down-regulated by Pa-PDT in all cell lines (Figure 1C).

### Cytotoxicity and ROS generation by miR-145-5p expression

**Figure 2:**
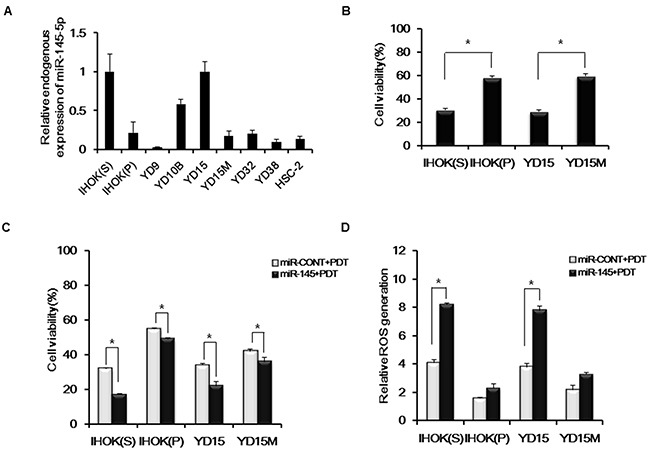
Cytotoxicity and ROS generation by miR-145-5p expression (**A**) Endogenous expression levels of miR-145-5p in IHOK and OSCC cells using real-time PCR. (**B**) Efficacy of Pa-PDT as a function of endogenous miR-145-5p expression. Cell viability was measured via MTT assay. (**C**) Cells were transfected with the miR-CONT or miR-145-5p mimic and then treated with PDT (0.3 μM, 0.5 J/cm^2^). Cell viability was measured via MTT assay. (**D**) After transfection with the miR-CONT or miR-145-5p mimic and incubation with 0.3 μM Pa for 2 h in a dark room, cells were gently rinsed with PBS and incubated with 10 μM of ROS dye at 37 °C and 5% CO_2_ for 20 min. After rinsing with PBS, cells were irradiated by light (0.5 J/cm^2^). After a 15 min incubation, the amount of ROS generated was quantified using flow cytometry. Data represent the median and 95% confidence interval of three independent samples, and the experiment was repeated three times per sample. Asterisks indicate statistical significance (* *p<*0.05).

To determine whether miR-145-5p has a major role in the efficacy of PDT, 2 types of IHOK cell lines and 7 oral cancer cell lines were screened for endogenous expression of miR-145-5p (Figure 2A). Regarding that each pair of IHOK cell lines and YD15 and YD15M (a metastatic cell line of YD15) cell lines showed a difference in expression of miR-145-5p, we selected them and examined their cell viabilities. The cell lines (IHOK(S) and YD15) showing higher endogenous expression of miR-145-5p revealed higher sensitivity to Pa-PDT than each paired cell line showing lower expression (Figure 2B). Upon treating a miR-145-5p mimic, cell viabilities of all 4 cell lines were significantly reduced (Figure 2C). ROS generation increased in all 4 cell lines after treating a miR-145-5p mimic. However, only IHOK(S) and YD15 cells, which showed higher miR-145-5p expression, had statistically significant differences in ROS generation (Figure 2D). These data suggested that the higher expression of miR-145-5p exerts the more potent PDT effect. Before PDT, the cell viability by transfection of a miR-145-5p mimic showed no difference in all 4 cell lines (data not shown).

### Induction of apoptosis by combined treatment of miR-145-5p mimic and PDT

**Figure 3:**
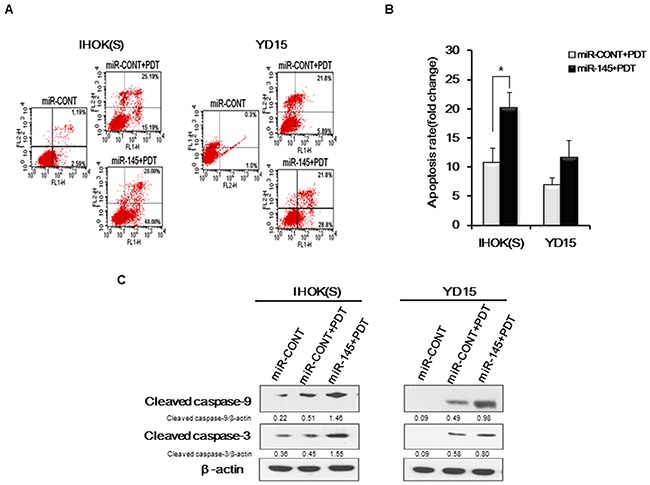
Apoptosis induction by combined treatment of miR-145-5p mimic and PDT **(A)** Cells were transfected with the miR-CONT or miR-145-5p mimic and then treated with Pa-PDT (0.3 μM, 0.5 J/cm^2^). After 4 h, cells were collected and double stained with annexin V/PI and analyzed using flow cytometry. (B) Cells were transfected with the miR-CONT or miR-145-5p mimic and then treated with Pa-PDT (0.3 μM, 0.5 J/cm^2^). Quantitative analysis indicated the population of apoptotic cells. Data represent the median and 95% confidence interval of three independent samples, and the experiment was repeated three times per sample. Asterisks indicate statistical significance (* *p<*0.05). (C) After 4 h, proteins were extracted from cells and resolved using 10% SDS-PAGE. Apoptosis-related proteins were detected using western blotting. Cropped gels retain suitable bands for each of antibodies. The relative intensity of protein expression was measured by Image J software.

To investigate whether the combined treatment of miR-145-5p mimic and Pa-PDT enhances apoptosis of cancer cells, we carried out annexin V/PI staining in IHOK(S) and YD15 cells. Compared to the treatment with miR-CONT, the number of apoptotic cells increased in both IHOK(S) and YD15 cells after combined treatment with the miR-145-5p mimic and Pa-PDT (Figure 3A and 3B). Apoptosis-related proteins, e.g., cleaved forms of caspase-9 and caspase-3, increased after treatment with the miR-145-5p mimic (Figure 3C), confirming that the high expression of miR-145-5p promotes apoptotic cell death in cancer cells by Pa-PDT. However, cleaved forms of caspase-9 showed no difference in YD15M after treatment with the miR-145-5p mimic (data not shown).

### RUNX3 activation by targeting of miR-145-5p

**Figure 4:**
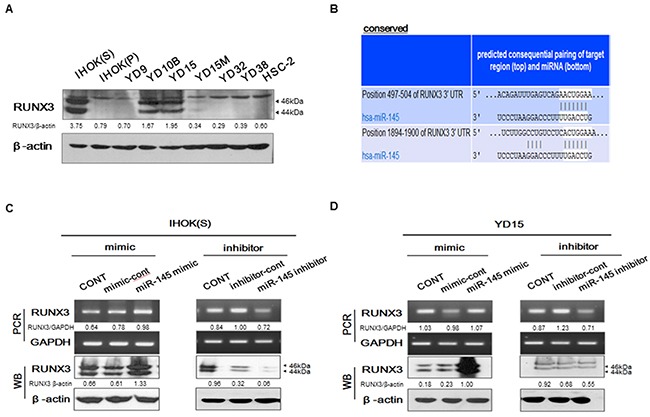
RUNX3 regulation by targeting of miR-145-5p (A) Endogenous protein levels of RUNX3 in IHOK and OSCC cells (**B**) Bioinformatics tools were used to predict the binding region of miR-145-5p in the RUNX3-3’UTR. (**C-D**) RUNX3 expression was positively regulated to transfection of miR-145-5p mimic(C) or inhibitor(D) in IHOK(S) and YD15. Data represent the mean±SD of three independent samples, and the experiment was repeated three times per sample (* *p<*0.05). Cropped gels retain suitable bands for each of antibodies. The relative intensity of protein expression was measured by Image J software.

Our previous study found that RUNX3 expression was proportional to Pa-PDT effect in OSCC cell lines, suggesting that RUNX3 might be a sensitive biomarker to determine the effect of Pa-PDT [17]. To validate whether miR-145-5p modulates RUNX3 expression, three miRNA target prediction algorithms (TargetScan, miRanda, and PicTar) were applied and putative binding sites for miR-145-5p were revealed in the RUNX3-3’UTR (Figure 4B). IHOK and OSCC cells were screened to examine endogenous expression of RUNX3 (Figure 4A). Corresponding to miR-145-5p expression, IHOK(S) and YD15 cells showed the highest expression level of RUNX3. To evaluate whether miR-145-5p activates RUNX3 expression, miR-145-5p mimic or inhibitor was transfected into IHOK(S) and YD15. The miR-145-5p mimic induced mRNA and protein expression of RUNX3 whereas miR-145-5p inhibitor suppressed mRNA and protein expression of RUNX3 in IHOK(S) and YD15 (Figure 4C and 4D). Before PDT, the cell viability by transfection of a miR-145-5p mimic or inhibitors showed no difference (data not shown).

### Proapoptotic bim upregulation by Pa-PDT

**Figure 5:**
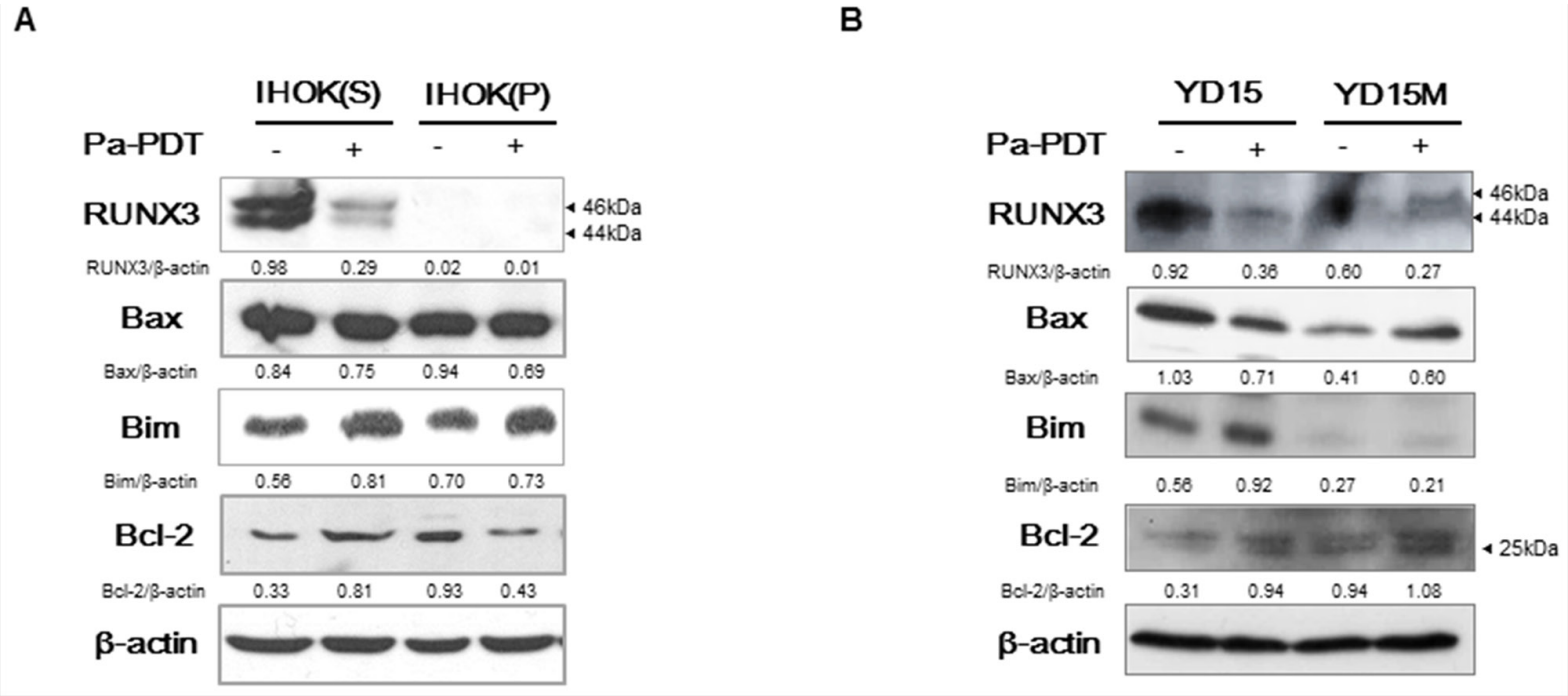
The expression of apoptosis-related genes through RUNX3 downstream by Pa-PDT (**A-B**) Cells were treated with Pa-PDT (0.3 μM Pa and 0.5 J/cm^2^). After 4 h, the cells were collected and the protein expression of RUNX3, Bax, Bim, and Bcl-2 were measured by western blotting. Cropped gels retain suitable bands for each of primers and antibodies. The relative intensity of protein expression was measured by Image J software.

To investigate the downstream pathway causing apoptosis in miR-145-5p-RUNX3 axis, the expression of several apoptosis-related proteins was examined. Like miR-145-5p, RUNX3 expression was reduced by Pa-PDT. In contrast, Bim expression increased by Pa-PDT, whereas the expression of Bax and Bcl-2 showed no difference (Figure 5A and 5B).

## DISCUSSION

PDT has been considered an effective treatment modality in many tumor types, and its effectiveness as a curative and palliative therapy is well documented, especially in the context of skin cancer. Numerous ongoing clinical studies have been designed to optimize PDT conditions. However, no standardized biological markers of cell death and PDT efficacy other than cell viability have been reported. Therefore, this study aimed to investigate whether apoptosis-related miRNA levels have an effect on PDT in OSCC.

We first examined whether miRNA expression was altered after Pa-PDT. With miRNA array-based expression analysis, several miRNAs were significantly down-regulated: the five miRNAs with the largest decreases were miR-32-5p, miR-143-3p, miR-145-5p, miR-338-3p, and miR-451a. Other miRNAs were up-regulated: the five miRNAs with the largest increases were miR-9-5p, miR-192-5p, miR-193a-5p, miR-204-5p, and miR-212-3p. The alteration of miRNA expression by PDT did not seem to be specific to Pa-PDT, as evidenced by Figure 1C. Only miR-145-5p was consistently down-regulated after Pa-PDT, lending support that miR-145-5p can be a target molecule to enhance PDT efficacy. The miR-145 has been acknowledged to be a tumor suppressor. The miR-145 is able to suppress cancer proliferation, invasion and metastases through suppression of mucin-1[18], RTKN[19], c-Myc[20], and EGFR[21].

To evaluate whether miR-145-5p enhances PDT efficacy in oral cancer, we selected two pairs of cell lines (IHOK(S) and IHOK(P), YD15 and YD15M), for each pair harbors less genetic variation than among cell lines from heterogenous origin. In our results, each pair of cell lines showed a difference in expression of miR-145-5p. We found that enhanced photosensitivity and ROS generation corresponded to increased expression of apoptosis-related miR-145-5p. In addition, the combined treatment of a miR-145-5p mimic and Pa-PDT elicited higher phototoxicity than the simple treatment of Pa-PDT. We also found that Pa-based PDT followed by transfection of a miR-145-5p mimic could significantly increase the induction of apoptosis. Ultimately, controlling miR-145-5p expression or activity could contribute to enhanced sensitivity of PDT in oral cancer.

To understand the mechanism why miR-145-5p expression enhances PDT efficacy in oral cancer cells, we attempted to see whether miR-145-5p can bind to RUNX3, which was found to be a biomarker to determine the sensitivity of PDT effect in OSCC in our previous study [17]. To investigate the mechanism of miR-145-5p in Pa-PDT efficacy, we used bioinformatics algorisms and found that RUNX3 mRNA 3’-UTR conserved eight- or seven-base oligonucleotide sites that match the miR-145-5p seed region. MicroRNAs have primarily been demonstrated to mediate posttranscriptional downregulation of expression, translation repression, and deadenylation-dependent decay of messages through partially complementary miRNA target sites in mRNA UTR. However, miRNAs and their associated protein complexes can additionally function to posttranscriptionally stimulate gene expression by direct and indirect mechanisms [22, 23]. In our study, to validate whether RUNX3 expression is regulated by targeting of miR-145-5p, miR-145-5p mimic or inhibitor was transfected. The miR-145-5p mimic transfection induced mRNA and protein expression of RUNX3, whereas the miR-145-5p inhibitor transfection reduced RUNX3 expression, suggesting that miR-145-5p positively activates RUNX3 expression. Taken together, the miR-145-5p expression enhances PDT efficacy *via* activating RUNX3 expression.

Accumulating evidences indicate that gene expression is regulated in a context-dependent, cell-type-specific manner. Corroborating this view, RUNX3 acts as tumor suppressor in gastro-intestinal cancers [24] and acts as an oncogene in head and neck cancer [25]. Likewise, widespread context dependency of miRNA-mediated regulation has been investigated, implying a much more complex post-transcriptional regulatory network than is currently known [26]. Given our results that the expression of miR-145-5p and RUNX3 was reduced by PDT, we could extrapolate that miR-145-5p and RUNX3 may also act as oncogene in oral cancer. In gastric cancer, RUNX3 cooperates with FoxO3a to participate in the induction of apoptosis by activating Bim [27]. Our data showed that Bim expression increased with reduced expression of miR-145 and RUNX3 by PDT. Considering that RUNX3 may cooperate with known or unknown cofactors maintaining its functional balance, Pa-PDT may break the functional balance of RUNX3 interacting with cofactors, inducing Bim expression, subsequently eventuating in apoptosis. However, considering context dependency of miRNA function, whether miR-145 may interact with other target sites by PDT should be further illuminated. Given the data that apoptotic cell death showed no difference in YD15M after treatment with the miR-145-5p mimic (data not shown), we extrapolate that the metastatic cell lines may interact with different target molecules from its original cell lines, eventuating in different outcome.

Taken together, the expression levels of apoptosis-related miRNAs were altered following Pa-PDT. The overexpression of miR-145-5p strengthens PDT efficacy. PDT has been limitedly applied to superficially located cancer, because of its potency and insufficient selectivity. To maximize the beneficial effects of PDT, the expression level of miR-145-5p can be a useful biomarker to predetermine patients’ sensitivity to PDT. Further, this study provides an insight to enhance the efficacy of Pa-PDT and serves as a new therapeutic strategy for effective treatment of oral cancer.

## MATERIALS AND METHODS

### Cell culture

We used 7 oral cancer cell lines (YD9, YD10B, YD32 and YD38 are OSCC cell lines; YD15 is a mucoepidermoid carcinoma cell line and YD15M is a metastatic cell line of YD15) [28] and 2 immortalized human oral keratinocyte (IHOK) cell lines for this study [17, 29]. IHOK cell lines were obtained by transfection of human papilloma virus (HPV) 16 E6/E7 DNA [29]. Two subtypes were obtained from the parent IHOK cell line based on morphology: IHOK(S) for spindle shape and IHOK(P) for polygonal shape [17]. Authentication of all cell lines was verified and shown to be free of contamination from other cell lines by Korean Cell Line Bank (KCLB). Cells were cultured in DMEM (Gibco BRL, USA) and Ham’s-F12 medium (Gibco BRL, USA) mixed in a 3:1 ratio and supplemented with 10% fetal bovine serum (FBS), 1% penicillin/streptomycin, 0.01 μg/ml cholera toxin, 0.04 μg/ml hydrocortisone, 0.5 μg/ml insulin, 0.5 μg/ml apo-transferrin, and 0.2 μg/ml 3’-5-triodo-1-thyroxine (Sigma, MO, USA). Cells were grown at 37 °C in a humidified incubator with 5% CO_2_.

### Photodynamic treatment

The Pa photosensitizer was obtained from Sigma (Sigma, MO, USA) and administered at 0.3 μM for 2 h. The irradiation dose was 0.5 J/cm^2^ [17].

### Cell viability

Cell viability was assessed using 3-(4, 5-dime-thylthiazol-2-yl)-2, 5-diphenyltetrazolium bromide (MTT) assay. Briefly, cells were seeded in a six-well plate (2×10^5^ cells/well), and MTT enzyme activities were measured. MTT solution (700 μl; Sigma, MO, USA) was added to each well, and cells were incubated for 3 h at 37 °C. After removing the MTT solution, 1 ml of DMSO (Sigma, MO, USA) was subsequently added to each well to dissolve the formazan dye. The optical density was measured at 540 nm with a micro-plate reader (Bio-Rad, CA, USA).

### miRNA micro-array

miRNA expression was profiled in YD10B (OSCC cell line), using miScript miRNA PCR Array (Qiagen, Hilden, Germany) in three independent experiments. Real-time quantitative PCR was performed with a LightCycler 480 II instrument and the manufacturer's software (Roche Applied Science, Mannheim, Germany). Gene Expression Suite software (Qiagen, Hilden, Germany) was used to process the array data. Automatic thresholds were checked individually and corrected when necessary.

### ROS generation

Intracellular levels of ROS were measured using the fluorescent probe 2’7’-dichlorofluorescein diacetate (H_2_DCFDA) (Molecular Probes, Inc., OR, USA) according to the manufacturer's instructions. Briefly, cells were treated with 0.3 μM Pa for 2 h in a dark room. Cells were gently rinsed with PBS and incubated with 10 μM of ROS dye at 37 °C for 20 min in the dark. Then, cells were gently rinsed with PBS and irradiated by light (0.5 J/cm^2^). Serum-free medium served as a negative control, and 10 μM H_2_O_2_ served as a positive control. After incubating for 15 min, ROS generation was measured using flow cytometry (Becton Dickinson, CA, USA), and Cell-Quest software (BD Biosciences, CA, USA) was used for data analysis.

### Apoptosis detection

Cells were treated with PDT (0.3 μM Pa and 0.5 J/cm^2^ light). After incubating for 4 h, cells were resuspended in binding buffer (Annexin V-FITC Apoptosis Detection Kit; BD Bioscience, CA, USA) at a concentration of 1×10^6^ cells/ml. Next, 4 μl of FITC annexin V and 4 μl of propidium iodide were added to 100 μl of cell suspension. After incubating in the dark for 15 min, 400 μl of binding buffer was added. Apoptosis was analyzed using flow cytometry (Becton Dickinson, CA, USA) using BD Cell-Quest software (BD Biosciences, CA, USA).

### Immunoblotting

Cells were treated with PDT, incubated for 4 h, then lysed (1.5×10^5^ cells) in cell lysis buffer (Cell Signaling Technology, MA, USA). Next, proteins were separated using 10% SDS-PAGE and transferred to a methanol-activated polyvinylidene fluoride membrane (Bio-Rad, CA, USA). The membrane was blocked for 1 h in PBST containing 5% milk and subsequently probed with anti-cleaved caspase-3 antibody, anti-cleaved caspase-9 antibody (Cell Signaling Technology, MA, USA), anti-RUNX3 (1:1,000) was obtained from Abcam (Abcam, Cambridge, UK). Additionally, antibodies against Bax (1:1,000), Bim (1:1,000) and Bcl-2 (1:1,000) were obtained from BioVision (BioVision, CA, USA). An anti-actin antibody (1:2,000) was used as a loading control (Sigma, MO, USA). Blots were then incubated for 1 h with horseradish peroxidase-conjugated anti-mouse (1:2,000) or rabbit (1:2,000) secondary antibodies (Cell Signaling Technology, MA, USA), and detection was done by chemiluminescence (Santa Cruz Biotechnology, CA, USA). The density was detected using chemiluminescence (Santa Cruz Biotechnology, CA, USA).

### Real-time PCR

Total RNA was extracted from whole cells and nucleoli using TRIzol Reagent and the RNeasy Kit (Qiagen, Hilden, Germany). To avoid DNA contamination, all samples were subjected to DNase treatment. In brief, 100 ng of purified total RNA were reverse transcribed, and quantification of specific miRNAs and U6 control transcripts was accomplished using the miScript SYBR Green PCR Kit (Qiagen, Hilden, Germany) with miScript Primer Assays HS-miR-9-5p (#MS00010752), Hs-miR-145-5p (#MS00003528), Hs-miR-192-5p (#MS00003689) and Hs-miR-338-3p (#MS00003990) according to the manufacturer's instructions. Amplification and detection of specific products were performed using a LightCycler 480 II instrument (Roche Applied Science, Mannheim, Germany) at 95 °C for 15 min, followed by 45 cycles of 94 °C for 15 s, 55 °C for 30 s, and 70 °C for 30 s. The threshold cycle (Ct) of each target gene was automatically defined, located in the liner amplification phase, and normalized against expression of the U6 control.

### Reverse transcriptase (RT)-PCR

Total RNA was isolated from each cell lysate using an RNeasy kit (Qiagen, Hilden, Germany), and cDNA was synthesized from 1 μg of the total RNA by using an Accu Power Hot Start PCR Pre Mix (Bioneer, Daejeon, South Korea) according to the manufacturers’ protocols. The following primers were used for RUNX3; forward (F): 5’-GGTACGGTGGTGACTGTGAT-3’, reverse (R): 5’-TGAACACACAGTGATGGTCAGG-3’, Bax; F: 5’-CCTTTTCTACTTTGTCAGCAA-3’, R: 5’-GAG GCCGTCCCAACCAC-3’, Bcl-2; F: 5’-GCCCTG TGGATGACTGAGTA-3’, R: 5’-ACTTGTGGCTCA GATAGGCA-3’, Bim; F: 5’-ATGGCAAAGCA ACCTTCT-3’, R: 5’-CGCATATCTGCAGGTTCA GCC-3’, GAPDH; F: 5’-GAAGGTGAAGGTCGGA GT-3’, R: 5’-GAAGATGGTGATGGGATTTC-3’. The reaction mixture was subjected to 30 cycles of 30 s at 94 °C, 30 s at 61 °C, and 45 s at 72 °C. The PCR products were visualized using ethidium bromide in 1% agarose-gel.

### miRNA transfection

Transfection of miRNA mimics is used to identify the targets and roles of particular miRNAs. In this protocol, cell seeding and transfection were performed on the same day. Shortly before transfection, cells (1.5×10^5^) were seeded in a 35-mm dish in 800 μl of an appropriate culture medium containing serum and antibiotics. The miR-145-5p mimic (#MSY0000437) and the miR-145-5p inhibitor (#MIN0000437) (Qiagen, Hilden, Germany) were diluted to a final concentration of 20 nM in 200 μl of culture medium without serum. Next, 3 μl of HiPerFect Transfection Reagent (Qiagen, Hilden, Germany) were mixed with the diluted mimic and inhibitor by vortexing. The samples were then incubated for 10 min at room temperature to allow forming transfection complexes. Subsequently, the complexes were added drop-wise to the cells. The transfected cells were cultured for 48 h before assay. The miRNA-control (miR-CONT) (#1027271) (Qiagen, Hilden, Germany) was used.

### Bioinformatics

Candidate RUNX3 targets were generated using the publicly available algorithms TargetScan (http://www.targetscan.org), miRanda (http://www.microrna.org), and PicTar (http://www.pictar.mdc-berline.de). For searching the binding region of miR-145-5p in the RUNX3-3’UTR, the “conserved” predictions were used in TargetScan.

### Statistical analysis

To determine statistically significant differences between measurements, Mann-Whitney *U* tests were performed. P values of <0.05 were considered significant. Unless otherwise stated, data presented were representative of three independent experiments.
